# Racial/Ethnic Disparities in Primary Care Quality Among Type 2 Diabetes Patients, Medical Expenditure Panel Survey, 2012

**DOI:** 10.5888/pcd13.160113

**Published:** 2016-08-04

**Authors:** Ruwei Hu, Leiyu Shi, Hailun Liang, Geraldine Pierre Haile, De-Chih Lee

**Affiliations:** Author Affiliations: Ruwei Hu, Department of Health Management, School of Public Health, Sun Yat-sen University, China, and Johns Hopkins Primary Care Policy Center, Baltimore, Maryland; Leiyu Shi, Johns Hopkins Primary Care Policy Center, Johns Hopkins Bloomberg School of Public Health, Baltimore, Maryland; Hailun Liang, Johns Hopkins Primary Care Policy Center, Baltimore, Maryland; Geraldine Pierre Haile, Johns Hopkins Primary Care Policy Center, Baltimore, Maryland, and Mathematica Policy Research, Oakland, California. Dr Lee is also affiliated with the Johns Hopkins Primary Care Policy Center, Baltimore, Maryland, and Da-Yeh University, Taiwan.

## Abstract

**Introduction:**

Racial and ethnic disparities exist in diabetes prevalence, access to diabetes care, diabetes-related complications and mortality rates, and the quality of diabetes care among Americans. We explored racial and ethnic disparities in primary care quality among Americans with type 2 diabetes.

**Methods:**

We analyzed data on adults with type 2 diabetes derived from the household component of the 2012 Medical Expenditure Panel Survey. Multiple regression and multivariate logistic regressions were used to examine the association between race/ethnicity and primary care attributes related to first contact, longitudinality, comprehensiveness, and coordination, and clusters of confounding factors were added sequentially.

**Results:**

Preliminary findings indicated differences in primary care quality between racial/ethnic minorities and whites across measures of first contact, longitudinality, comprehensiveness, and coordination. After controlling for confounding factors, these differences were no longer apparent; all racial/ethnic categories showed similar rates of primary care quality according to the 4 primary care domains of interest in the study.

**Conclusion:**

Results indicate equitable primary care quality for type 2 diabetes patients across 4 key domains of primary care after controlling for socioeconomic characteristics. Additional research is necessary to support these findings, particularly when considering smaller racial/ethnic groups and investigating outcomes related to diabetes.

## Introduction

Approximately 29.1 million Americans have diabetes, and type 2 diabetes accounts for 90% to 95% of all diagnosed cases in adults (1). An estimated 1 in 4 people with diabetes are unaware of their condition (1). In 2010, diabetes was the seventh leading cause of death in the United States, and the total direct and indirect costs associated with the condition were an estimated $245 billion (1). Even after adjusting for age and sex differences at the population level, average medical expenditures among people diagnosed with diabetes were 2.3 times higher than among people without diabetes (1).

Decades of literature have found racial and ethnic disparities in the prevalence of diabetes, access to diabetes care, diabetes-related complications and mortality rates, and the quality of diabetes care (2). More specifically, the risk of diabetes was 77% higher among African Americans and 66% higher among Hispanic/Latino Americans than among non-Hispanic white Americans (3). Non-Hispanic black patients with diabetes were significantly less likely to receive low-density lipoprotein (LDL) cholesterol testing than were white patients who received care at the same facility (4). Black patients were also more likely than white patients to have poor blood pressure and cholesterol control (4,5).

Although previous literature describes racial disparities in diabetes prevalence and treatment, little exploration has been conducted on the relationship between race and ethnicity and primary care quality among patients with diabetes. This is relevant, because primary care is effective in the management of diabetes (6–8). Assessments of the quality of primary care should consider the 4 cardinal dimensions: first contact, longitudinality, comprehensiveness, and coordination (9). First contact care refers to care first sought from the primary care provider when a new health or medical need arises. Longitudinality refers to the longitudinal use of a regular source of care over time, regardless of the presence or absence of disease or injury. Comprehensiveness refers to the availability of a range of services in primary care and their provision by a primary care provider across the spectrum of needs for all but the most uncommon problems in the population. Coordination is the linking of health care visits and services so that patients receive appropriate care for all their health problems, physical as well as mental (10).

 It was difficult to draw firm conclusions from the literature about racial/ethnic differences in primary care quality among type 2 diabetes patients (11–14). For example, results from a study conducted by Brown et al found that being from a racial/ethnic minority group was not consistently associated with worse processes or outcomes in quality of diabetes care, and not all differences favored white patients (11). Results from another study by Murray-García et al suggested that Asian and Latino patients were less satisfied with primary care than were white patients (12). In addition, many previous studies included only clinical measures — such as hemoglobin A1c, LDL cholesterol level, and plasma glucose — as their research outcomes of interest (11,13–15). Other studies on patients’ ratings of quality of care included samples only from a specific geographic area or setting (eg, using state and health systems data) (11,12).

To our knowledge, little nationwide and updated research exists that focuses on the racial/ethnic disparities in primary care quality among type 2 diabetes patients. The purpose of our study was to explore racial/ethnic disparities in primary care quality — particularly, the domains of first contact, longitudinality, comprehensiveness, and coordination — among Americans with type 2 diabetes.

## Methods

We used data from the household component of the 2012 Medical Expenditure Panel Survey (MEPS), which was the latest publicly accessible data set at the time of our study. MEPS is a nationally representative survey of the US noninstitutionalized civilian population, comprising survey data of families and individuals and their medical providers and employers. MEPS is supported by the Agency for Healthcare Research and Quality (16). The 2012 MEPS contained 38,974 observations; our study included respondents aged 18 or older who reported being told by a clinician that they had type 2 diabetes. We excluded respondents who had missing values for race/ethnicity. A total of 2,617 people with type 2 diabetes were included in the study, representing an estimated 21,813,781 adults with self-reported diabetes. The final sample and population size for each dependent variable varied because of missing or inapplicable values for different dependent measures.

### Measures

The household component of MEPS collects detailed data on demographic characteristics, health conditions, health status, use of medical care services, charges and payments, access to primary care, satisfaction with care, health insurance coverage, annual income, and employment. In this study, measures of primary care attributes (dependent variables), race/ethnicity (independent variable), and individual characteristics (covariates) were used.

According to previous work conducted on primary care (17–19), we considered 4 cardinal domains of primary care — first contact, longitudinality, comprehensiveness, and coordination — in looking at primary care attributes as dependent variables of interest in the analyses. Eight questions were selected from MEPS pertaining to first contact and were coded. First contact characteristics were have a usual source of care (USC) (yes = 1, no = 0), because research has used “has a USC” as a structural component of the health care system that appropriately reflects an individual’s entry into the system (12); provider type of USC (facility = 0, person/person in facility = 1); provider specialty of USC (primary care = 1, other = 0); USC location (office = 1, hospital = 0); difficulty contacting USC by telephone (not very difficult = 1, very difficult = 0); USC has office hours on nights/weekends (yes = 1, no = 0); time to get to USC (≤30 min = 1, >30 min = 0); and difficulty in getting to USC (not difficult = 1, difficult = 0). Longitudinality was assessed with 1 question: “Does the USC provider listen?” (yes = 1, no = 0). Comprehensiveness was assessed with 1 question: “Does the patient go to a USC for preventive health care?” (yes = 1, no = 0). Coordination was assessed with 2 questions: “Does the provider ask about other treatments?” (yes = 1, no = 0) and “Does the patient go to a USC for referrals?” (yes = 1, no = 0).

Andersen and Aday’s access-to-care framework, among the most well-known and widely used models for demonstrating the factors that lead to the use of health care services, was used in the selection of individual covariates that may be related to the experience of primary care (20). According to this framework, health care use is influenced by predisposing, enabling, and need factors. Predisposing factors refers to exogenous factors — such as age, sex, race/ethnicity, and other social structure and health belief factors — that affect one’s inclination to use health care services. Enabling factors are the ability of an individual to access care and the availability of services. Need factors take into account factors such as health status, existing diseases, and other chronic conditions (20). On the basis of these components of the framework, we extracted covariate measures for the study. Predisposing characteristics were age (18−45 y, 46−64 y, >64 y); sex (male, female); race/ethnicity (non-Hispanic white, non-Hispanic black, Hispanic, non-Hispanic Asian, non-Hispanic other); health insurance (public, private, no insurance); education (no degree, high school diploma, bachelor and higher degree, other); employment status (not employed, employed); annual income (<$20,000, $20,000–$39,999, ≥$40,000); and marital status (married, not married). Enabling factors included were metropolitan statistical area (MSA), a geographical region with a high population density at its core and close economic ties (yes, no); and census region (Northeast, Midwest, South, West). Need factors of interest were perceived health status (excellent/very good/good, fair/poor); perceived mental health status (excellent/very good/good, fair/poor); need help with activities of daily living (ADL help screener) (yes, no); and need help with instrumental activities of daily living (IADL help screener) (yes, no).

### Analysis

Data analysis was performed using Stata/SE version 14.0 (StataCorp, LP), accounting for the multistage, stratified cluster sampling associated with MEPS. All analysis accounted for sampling weights. Bivariate comparisons were performed between an individual’s race/ethnicity and primary care attributes. We used χ^2^ tests to determine whether there were differences among racial/ethnic groups in primary care quality. We also constructed 4 scored summary variables representing each of the primary care domains. We first assigned scores to each of the questions and then created an integrated variable by summing the total scores of questions within each domain. Therefore, the score ranges for the 4 domains — first contact, longitudinality, comprehensiveness, and coordination — were 0 to 8, 0 to 1, 0 to 1, and 0 to 2, respectively. However, we recoded the coordination domain as dichotomous because of the distribution of total scores within the domain. We recoded the coordination domain score as 0 if the original score was 0 or 1, and we recoded the domain score as 1 if the original score was 2. ANOVA was performed to determine whether there was significant difference among racial/ethnic groups in first contact quality. We used χ^2^ tests to determine whether there were differences among racial/ethnic groups in longitudinality, comprehensiveness, and coordination domains. Multiple regression and multivariate logistic regressions were used to examine the association between race/ethnicity and primary care attributes related to first contact, longitudinality, comprehensiveness, and coordination, by sequentially adding the clusters of confounding factors in the following order: predisposing factors, enabling factors, and need factors. In our regression models, we used the 4 integrated variables of 4 primary care domains rather than individual items and applied hierarchical modeling approach to investigate in greater depth, so we could understand the contributors to disparities in primary care quality. Model 1 was unadjusted regressions. Model 2 was adjusted for predisposing factors. Model 3 was adjusted for predisposing and enabling factors. Model 4 was fully adjusted, accounting for predisposing, enabling, and need factors. We used standard errors, *P* values, β coefficients, odds ratios (ORs), and 95% confidence intervals (CIs) to interpret significance and effect size.

## Results

In 2012, 21,813,781 American adults had type 2 diabetes. Most of those with the condition were aged 46 to 64 years (43.8%); 42.8% were aged 65 or older. Adults with a high school diploma, the unemployed, and those with annual income below $20,000 accounted for more than half of those with diabetes. The southern census region represented 41% of diabetes cases; urban areas accounted for 82% (Table 1).

**Table 1:** Demographic and Primary Care Characteristics for Population With Type 2 Diabetes, Medical Expenditure Panel Survey, 2012

Characteristic	No. (N = 2,617)	Weighted No. (N = 21,813,781) (Weighted %) [95% CI]
**Predisposing Factors**		
**Age, y^a^**		
18–45	403	2,924,379 (13.4) [11.7–15.1]
46–64	1,164	9,561,753 (43.8) [40.9–46.7]
>64	1,049	9,325,508 (42.8) [39.9–45.7]
**Sex^b^**		
Male	1,230	11,156,923 (51.1) [48.7–53.5]
Female	1,387	10,656,858 (48.9) [46.5–51.3]
**Race/ethnicity^a^**		
Non-Hispanic white	953	13,159,194 (60.3) [57.0–63.6]
Non-Hispanic black	683	3,368,274 (15.4) [13.2–17.6]
Hispanic	743	3,592,967 (16.5) [13.8–19.2]
Non-Hispanic Asian	171	1,012,791 (4.6) [3.5–5.7]
Non-Hispanic other	67	680,554 (3.1) [1.9–4.3]
**Health insurance^a^**		
Private	1,260	12,567,988 (57.6) [54.7–60.5]
Public	1,041	7,514,538 (34.5) [31.8–37.2]
No insurance	316	1,731,254 (7.9) [6.6–9.2]
**Education^a^**		
No degree	373	2,135,699 (20.9) [18.4–23.4]
High school diploma	655	5,823,750 (57.0) [53.5–60.5]
≥Bachelor degree	193	1,686,156 (16.5) [14.0–19.0]
Other	63	565,057 (5.5) [3.8–7.2]
**Employment status^a^**		
Not employed	1,566	12,807,108 (58.9) [56.2–61.6]
Employed	1,046	8,952,016 (41.1) [38.4–43.8]
**Annual income, $**		
<20,000	1,500	10,985,633 (50.4) [47.7–53.1]
20,000−39,999	612	5,322,165 (24.4) [22.4–26.4]
≥40,000	504	5,493,176 (25.2) [22.7–27.7]
**Marital status^a^**		
Not married	1,245	9,458,394 (43.4) [41.0–45.8]
Married	1,372	12,355,387 (56.6) [54.2–59.0]
**Enabling Factors**		
**Metropolitan statistical area^b^**		
No	387	3,870,681 (17.7) [14.6–20.8]
Yes	2,229	17,940,960 (82.3) [79.2–85.4]
**Census region**		
Northeast	424	3,855,015 (17.7) [15.2–20.2]
Midwest	449	4,705,831 (21.6) [19.1–24.1]
South	1,109	8,893,429 (40.8) [38.1–43.5]
West	634	4,357,367 (20.0) [17.8–22.2]
**Need Factors**		
**Perceived health status^a^**		
Excellent/very good/good	1,656	14,350,075 (65.8) [63.4–68.2]
Fair/poor	961	7,463,705 (34.2) [31.8–36.6]
**Perceived mental health status^a^**		
Excellent/very good/good	2,203	18,510,339 (84.9) [83.1–86.7]
Fair/poor	414	3,303,441 (15.1) [13.3–16.9]
**Help with ADL^a^**		
No	2,457	20,608,010 (94.5) [93.2–95.8]
Yes	160	1,205,770 (5.5) [4.2–6.8]
**Help with IADL^a^**		
No	2,362	19,750,771 (90.5) [89.1–91.9]
Yes	255	2,063,009 (9.5) [8.1–10.9]
**Primary Care Attribute**		
**First Contact**		
**Have USC^a^**		
No	253	1,676,213 (7.8) [6.3–9.3]
Yes	2,316	19,799,721 (92.2) [90.7–93.7]
**Provider type of USC^a^**		
Facility	1,140	8,879,718 (44.9) [41.5–48.3]
Person/person in facility	1,176	10,920,003 (55.1) [51.7–58.5]
**Provider specialty of USC**		
Primary care	1,066	9,727,524 (89.1) [86.1–92.1]
Other	110	1,192,479 (10.9) [7.9–13.9]
**USC location**		
Office	1,664	15,321,478 (77.5) [74.7–80.3]
Hospital	648	4,453,434 (22.5) [19.7–25.3]
**Difficulty in contacting USC by telephone^a^**		
Not very difficult	2,096	17,845,798 (93.9) [92.5–95.3]
Very difficult	139	1,148,599 (6.1) [4.7–7.5]
**USC has office hours nights/weekends^a^**		
No	1,412	12,252,897 (68.9) [66.1–71.7]
Yes	670	5,528,129 (31.1) [28.3–33.9]
**How long it takes to get to USC^a^**		
≤30 min	1,974	17,095,937 (86.4) [84.5–88.3]
>30 min	338	2,684,740 (13.6) [11.7–15.5]
**How difficult is it to get to USC**		
Difficult	2,280	19,579,459 (99.0) [98.5–99.5]
Not difficult	31	192,250 (1.0) [0.5–1.5]
**Longitudinality**		
**USC provider listens**		
No	28	151,190 (0.8) [0.4–1.2]
Yes	2,139	18,552,573 (99.2) [98.8–99.6]
**Comprehensiveness**		
**Goes to USC for preventive health care^a^**		
No	24	155,285 (0.8) [0.4–1.2]
Yes	2,288	19,605,865 (99.2) [98.8–99.6]
**Coordination**		
**Provider asks about other treatments**		
No	379	3,188,383 (16.6) [14.5–18.7]
Yes	1,875	16,007,106 (83.4) [81.3–85.5]
**Goes to USC for referrals**		
No	35	369,907 (1.9) [1.1–2.7]
Yes	2,277	19,416,969 (98.1) [97.3–98.9]

Abbreviation: ADL, activities of daily living; CI, confidence interval; IADL, instrumental activities of daily living; USC, usual source of care.

a *P* < .001.

b *P* < .01.

When looking at primary care characteristics among the population with diabetes by race/ethnicity, 91% of non-Hispanic blacks, 86% of Hispanics, and 89% of non-Hispanic other racial/ethnic groups reported having a USC, compared with 94% of non-Hispanic whites and 94% of non-Hispanic Asians (*P* < .01) (Table 2). Non-Hispanic blacks (49%), Hispanics (60%), and non-Hispanic others (60%) were more likely to report a facility rather than a specific provider to be their USC than were white (40%) and Asian adults (38%) (*P* < .001); hospitals accounted for 25% of USC locations among non-Hispanic blacks, 43% among Hispanics, 25% among non-Hispanic others, and only 17% and 18% among non-Hispanic whites and Asians, respectively (*P* < .001). No significant differences were found in mean scores for first contact domain among the 5 racial/ethnic groups. Non-Hispanic others reported going to a USC for preventive health care at a lower rate (96%) than did Hispanics and non-Hispanic white, black, and Asian adults, who all reported approximately 99% on this comprehensiveness item (*P* < .05). A similar pattern emerged when looking at whether individuals sought care at a USC for referrals, where non-Hispanic others reported 91%, compared with 98% to 99% for the other racial/ethnic groups (*P* < .01). The results for the recoded measure of evaluating the coordination domain were also significantly different among the 5 racial/ethnic groups; 82% of non-Hispanic blacks, 83% of Hispanics, 74% of non-Hispanic Asians, and 69% of non-Hispanic others reported as having primary care coordination, compared with 83% of non-Hispanic whites (*P* < .05) (Table 2).

**Table 2:** Primary Care Characteristics for Population With Type 2 Diabetes, by Race/Ethnicity, Medical Expenditure Panel Survey, 2012

Characteristic	Race/Ethnicity														
	Non-Hispanic White			Non-Hispanic Black			Hispanic			Non-Hispanic Asian			Non-Hispanic Other		
	n	Wt %	SE	n	Wt %	SE	n	Wt %	SE	n	Wt %	SE	n	Wt %	SE
**First Contact**															
**Have USC^a^**															
No	55	5.86	0.96	63	8.89	1.3	120	13.93	1.7	11	5.59	2	4	11.1	7
Yes	886	94.14	0.96	605	91.11	1.3	610	86.07	1.7	153	94.41	2	62	88.9	7
**Provider type of USC^b^**															
Facility	358	39.67	2.3	302	49.1	2.9	382	60.44	3	64	38.39	4.6	34	60.06	9
Person/person in facility	528	60.33	2.3	303	50.9	2.9	228	39.56	3	89	61.61	4.6	28	39.94	9
**Provider specialty of USC**															
Primary care	469	88.11	2	277	91.26	2	215	92.48	2.5	80	89.47	4.1	25	87.12	8.5
Other	59	11.89	2	26	8.74	2	13	7.52	2.5	9	10.53	4.1	3	12.88	8.5
**USC location^b^**															
Office	725	82.96	1.9	441	74.62	2.3	336	57.39	3.1	117	81.79	3.5	45	75.14	5.7
Hospital	160	17.04	1.9	161	25.38	2.3	274	42.61	3.1	36	18.21	3.5	17	24.86	5.7
**Difficulty in contacting USC by telephone**															
Not very difficult	798	93.93	1.1	560	96.57	0.82	545	91.17	1.9	134	94.05	2.5	59	95.41	3.1
Very difficult	54	6.07	1.1	22	3.43	0.82	52	8.83	1.9	8	5.95	2.5	3	4.59	3.1
**USC has office hours nights/weekends**															
No	554	70.03	2	380	70.02	2.4	370	68.97	2.5	78	58.66	5.7	30	55.22	8.1
Yes	237	29.97	2	170	29.98	2.4	182	31.03	2.5	59	41.34	5.7	22	44.78	8.1
**How long it takes to get to USC**															
≤30 min	769	87.67	1.3	501	83.81	1.7	518	84.89	1.9	135	88.13	2.9	51	79.26	7.2
>30 min	116	12.33	1.3	102	16.19	1.7	91	15.11	1.9	18	11.87	2.9	11	20.74	7.2
**How difficult is it to get to USC**															
Difficult	875	99.26	0.3	594	98.43	0.63	596	98.2	0.6	153	100	0	62	100	0
Not difficult	10	0.74	0.3	8	1.57	0.63	13	1.8	0.6	NA	NA	NA	NA	NA	NA
**First contact domain: n, mean score, SE**	480	6.77	0.05	282	6.82	0.06	219	6.74	0.07	80	7.01	0.10	20	6.59	0.17
**Longitudinality**															
**USC provider listens^c^**															
No	4	0.4	0.21	9	1.52	0.58	13	1.86	0.6	2	1.08	0.8	NA	NA	NA
Yes	840	99.6	0.21	564	98.48	0.58	555	98.14	0.6	122	98.92	0.8	58	100	0
**Comprehensiveness**															
**Goes to USC for preventive health care^c^**															
No	5	0.51	0.25	6	1.03	0.44	9	1.08	0.3	2	0.81	0.59	2	3.74	2.6
Yes	879	99.49	0.25	598	98.97	0.44	600	98.92	0.3	151	99.19	0.59	60	96.26	2.6
**Coordination**															
**Provider asks about other treatments**															
No	136	15.54	1.6	98	17.39	1.7	101	16.15	2.2	31	25.51	4	13	22.81	7.4
Yes	722	84.46	1.6	489	82.61	1.7	498	83.85	2.2	118	74.49	4	48	77.19	7.4
**Goes to USC for referrals^a^**															
No	14	1.93	0.55	7	1.29	0.5	9	1.29	0.5	1	0.51	0.52	4	8.6	5
Yes	872	98.07	0.55	597	98.71	0.5	598	98.71	0.5	152	99.49	0.52	58	91.4	5
**Coordination domain^c^**															
No	146	16.65	0.02	109	18.27	0.02	105	17.18	0.02	38	25.51	0.04	19	30.78	0.07
Yes	732	83.35	0.02	490	81.73	0.02	504	82.82	0.02	112	74.49	0.04	43	69.22	0.07

Abbreviations: NA, not applicable; SE, standard error; USC, usual source of care; Wt %, weighted percentage.

a *P* < .01.

b *P* < .001.

c *P* < .05.

Table 3 shows the results of cumulative effect of factors on racial/ethnic differences in primary care characteristics for adults with type 2 diabetes at the population level. Model 1 shows the unadjusted β coefficient or OR for each primary care domain among each racial/ethnic minority group compared with whites. Similar to the finding of the first contact domain score in Table 2 — except for non-Hispanic Asians, who were more likely to rate higher (β = 0.264; 95% CI, 0.075−0.453; *P* < .01) — no other significant associations were found among racial/ethnic groups and differences in the first contact scores. For longitudinality indicators, non-Hispanic blacks (OR = 0.298; 95% CI, 0.091−0.974; *P* < .05) and Hispanics (OR = 0.203; 95% CI, 0.066−0.627; *P* < .01) were less likely to report that their USC providers listened to them compared with non-Hispanic whites. With respect to comprehensiveness indicators, non-Hispanic others (OR = 0.172; 95% CI, 0.033−0.905; *P* < .05) were less likely than whites to report that they went to their USC for preventive health care.

**Table 3:** Regression Models for Cumulative Effect of Different Factors on Racial/Ethnic Differences in Primary Care Characteristics for Population With Diabetes, Medical Expenditure Panel Survey, 2012

Characteristic	Model 1: Unadjusted	Model 2: Model 1 + Predisposing Factors	Model 3: Model 2 + Enabling Factors	Model 4: Model 3 + Need Factors
**First Contact β (95% CI)**				
**Race/ethnicity**				
Non-Hispanic white	1 [Reference]	1 [Reference]	1 [Reference]	1 [Reference]
Non-Hispanic black	0.049 (−0.069 to 0.167)	0.117 (−0.004 to 0.238)	0.078 (−0.046 to 0.201)	0.070 (−0.054 to 0.194)
Hispanic	0.011 (−0.118 to 0.141)	0.046 (−0.088 to 0.181)	0.027 (−0.114 to 0.168)	0.025 (−0.116 to 0.167)
Non-Hispanic Asian	0.264^a^ (0.075 to 0.453)	0.258^a^ (0.066 to 0.450)	0.270^a^ (0.070 to 0.469)	0.269^a^ (0.069 to 0.469)
Non-Hispanic other	−0.062 (−0.413 to 0.289)	−0.089 (−0.439 to 0.260)	−0.064 (−0.412 to 0.285)	−0.057 (−0.407 to 0.292)
**Predisposing Factors**				
**Age, y**				
18–45		1 [Reference]	1 [Reference]	1 [Reference]
46–64		−0.187^b^ (−0.353 to −0.022)	−0.188^b^ (−0.353 to −0.023)	−0.182^b^ (−0.347 to −0.017)
>64		−0.086 (−0.259 to 0.088)	−0.079 (−0.251 to 0.094)	−0.082 (−0.255 to 0.091)
**Sex**				
Male		1 [Reference]	1 [Reference]	1 [Reference]
Female		0.058 (−0.042 to 0.159)	0.056 (−0.045 to 0.156)	0.053 (−0.048 to 0.153)
**Health insurance**				
Private		1 [Reference]	1 [Reference]	1 [Reference]
Public		0.010 (−0.113 to 0.133)	0.010 (−0.112 to 0.132)	0.022 (−0.101 to 0.146)
No insurance		−0.092 (−0.320 to 0.137)	−0.082 (−0.310 to 0.145)	−0.090 (−0.317 to 0.138)
**Education**				
No degree		1 [Reference]	1 [Reference]	1 [Reference]
High school diploma		0.016 (−0.149 to 0.180)	0.000 (−0.163 to 0.164)	0.004 (−0.160 to 0.168)
≥Bachelor degree		−0.051 (−0.276 to 0.174)	−0.079 (−0.303 to 0.145)	−0.082 (−0.306 to 0.143)
Other		−0.155 (−0.487 to 0.178)	−0.150 (−0.481 to 0.181)	−0.145 (−0.477 to 0.187)
**Employment status**				
Not employed		1 [Reference]	1 [Reference]	1 [Reference]
Employed		0.029 (−0.107 to 0.165)	0.034 (−0.101 to 0.170)	0.022 (−0.116 to 0.159)
**Annual income, $**				
<20,000		1 [Reference]	1 [Reference]	1 [Reference]
20,000–39,999		0.138^b^ (0.007 to 0.270)	0.141^b^ (0.009 to 0.273)	0.140^b^ (0.007 to 0.273)
≥40,000		0.106 (−0.058 to 0.270)	0.094 (−0.071 to 0.259)	0.091 (−0.074 to 0.257)
**Marital status**				
Not married		1 [Reference]	1 [Reference]	1 [Reference]
Married		0.203^c^ (0.098 to 0.308)	0.199^c^ (0.094 to 0.304)	0.189^c^ (0.084 to 0.295)
**Enabling Factors**				
**Metropolitan statistical area**				
No			1 [Reference]	1 [Reference]
Yes			0.164^b^ (0.031 to 0.296)	0.160^b^ (0.028 to 0.293)
**Census region**				
Northeast			1 [Reference]	1 [Reference]
Midwest			−0.169^b^ (−0.333 to −0.004)	−0.165 (−0.330 to 0.000)
South			−0.085 (−0.225 to 0.055)	−0.080 (−0.221 to 0.061)
West			−0.280^a^ (−0.441 to −0.120)	−0.274^a^ (−0.436 to −0.112)
**Need Factors**				
**Perceived health status**				
Excellent/very good/good				1 [Reference]
Fair/poor				0.017 (−0.102 to 0.136)
**Perceived mental health status**				
Excellent/very good/good				1 [Reference]
Fair/poor				−0.083 (−0.240 to 0.074)
**Help with ADL**				
No				1 [Reference]
Yes				0.054 (−0.171 to 0.280)
**Help with IADL**				
No				1 [Reference]
Yes				−0.117 (−0.316 to 0.082)
**Longitudinality, OR (95% CI)**				
**Race/ethnicity**				
Non-Hispanic white	1 [Reference]	1 [Reference]	1 [Reference]	1 [Reference]
Non-Hispanic black	0.298^b^ (0.091 to 0.974)	0.329 (0.098 to 1.110)	0.386 (0.112 to 1.332)	0.384 (0.111 to 1.327)
Hispanic	0.203^a^ (0.066 to 0.627)	0.270^b^ (0.083 to 0.883)	0.361 (0.107 to 1.224)	0.365 (0.107 to 1.245)
Non-Hispanic Asian	0.290 (0.053 to 1.603)	0.343 (0.060 to 1.980)	0.487 (0.080 to 2.969)	0.464 (0.075 to 2.854)
Non-Hispanic other	NA	NA	NA	NA
**Predisposing Factors**				
**Age, y**				
18–45		1 [Reference]	1 [Reference]	1 [Reference]
46–64		2.323 (0.938 to 5.756)	2.430 (0.977 to 6.043)	2.324 (0.931 to 5.798)
>64		3.267^b^ (1.129 to 9.451)	3.404^b^ (1.179 to 9.830)	3.303^b^ (1.134 to 9.618)
**Sex**				
Male		1 [Reference]	1 [Reference]	1 [Reference]
Female		2.205 (0.990 to 4.910)	2.258^b^ (1.012 to 5.035)	2.368^b^ (1.059 to 5.295)
**Health insurance**				
Private		1 [Reference]	1 [Reference]	1 [Reference]
Public		0.579 (0.210 to 1.598)	0.615 (0.222 to 1.705)	0.645 (0.231 to 1.806)
No insurance		1.141 (0.279 to 4.676)	1.032 (0.251 to 4.240)	1.124 (0.270 to 4.684)
**Education**				
No degree		1 [Reference]	1 [Reference]	1 [Reference]
High school diploma		0.477 (0.117 to 1.945)	0.470 (0.115 to 1.928)	0.464 (0.112 to 1.911)
≥Bachelor degree		0.740 (0.072 to 7.625)	0.772 (0.074 to 8.089)	0.765 (0.073 to 8.071)
Other		0.210 (0.020 to 2.244)	0.221 (0.021 to 2.371)	0.214 (0.020 to 2.325)
**Employment status**				
Not employed		1 [Reference]	1 [Reference]	1 [Reference]
Employed		0.891 (0.320 to 2.477)	0.840 (0.295 to 2.392)	0.845 (0.290 to 2.463)
**Annual income, $**				
<20,000		1 [Reference]	1 [Reference]	1 [Reference]
20,000–39,999		1.632 (0.522 to 5.104)	1.701 (0.539 to 5.362)	1.702 (0.534 to 5.420)
≥40,000		3.266 (0.602 to 17.704)	3.656 (0.664 to 20.134)	3.621 (0.660 to 19.865)
**Marital status**				
Not married		1 [Reference]	1 [Reference]	1 [Reference]
Married		0.655 (0.282 to 1.519)	0.634 (0.271 to 1.481)	0.620 (0.264 to 1.458)
**Enabling Factors**				
**Metropolitan statistical area**				
No			1 [Reference]	1 [Reference]
Yes			0.834 (0.237 to 2.939)	0.828 (0.234 to 2.925)
**Census region**				
Northeast			1 [Reference]	1 [Reference]
Midwest			NA	NA
South			1.096 (0.370 to 3.246)	1.130 (0.379 to 3.371)
West			0.890 (0.280 to 2.831)	0.932 (0.290 to 2.997)
**Need Factors**				
**Perceived health status**				
Excellent/very good/good				1 [Reference]
Fair/poor				0.598 (0.260 to 1.378)
**Perceived mental health status**				
Excellent/very good/good				1 [Reference]
Fair/poor				2.540 (0.665 to 9.707)
**Help with ADL**				
No				1 [Reference]
Yes				0.813 (0.104 to 6.336)
**Help with IADL**				
No				1 [Reference]
Yes				0.929 (0.165 to 5.245)
**Comprehensiveness, OR (95% CI)**				
**Race/ethnicity**				
Non-Hispanic white	1 [Reference]	1 [Reference]	1 [Reference]	1 [Reference]
Non-Hispanic black	0.567 (0.172 to 1.866)	0.664 (0.194 to 2.277)	0.664 (0.187 to 2.363)	0.688 (0.192 to 2.462)
Hispanic	0.377 (0.126 to 1.131)	0.468 (0.142 to 1.537)	0.535 (0.156 to 1.831)	0.527 (0.153 to 1.822)
Non-Hispanic Asian	0.422 (0.081 to 2.193)	0.406 (0.075 to 2.196)	0.504 (0.089 to 2.861)	0.482 (0.084 to 2.766)
Non-Hispanic other	0.172^b^ (0.033 to 0.905)	0.201 (0.037 to 1.105)	0.221 (0.040 to 1.230)	0.209 (0.037 to 1.189)
**Predisposing Factors**				
**Age, y**				
18–45		1 [Reference]	1 [Reference]	1 [Reference]
46–64		2.599 (0.847 to 7.980)	2.603 (0.847 to 7.998)	2.594 (0.838 to 8.029)
>64		1.272 (0.389 to 4.158)	1.270 (0.388 to 4.156)	1.262 (0.380 to 4.193)
**Sex**				
Male		1 [Reference]	1 [Reference]	1 [Reference]
Female		1.680 (0.718 to 3.929)	1.732 (0.741 to 4.049)	1.733 (0.739 to 4.063)
**Health insurance**				
Private		1 [Reference]	1 [Reference]	1 [Reference]
Public		0.777 (0.257 to 2.352)	0.807 (0.266 to 2.448)	0.802 (0.261 to 2.464)
No insurance		0.295 (0.080 to 1.092)	0.281 (0.076 to 1.039)	0.300 (0.081 to 1.119)
**Education**				
No degree		1 [Reference]	1 [Reference]	1 [Reference]
High school diploma		1.761 (0.446 to 6.955)	1.721 (0.433 to 6.831)	1.736 (0.435 to 6.926)
≥Bachelor degree		0.893 (0.151 to 5.271)	0.908 (0.151 to 5.446)	0.897 (0.149 to 5.416)
Other		0.562 (0.058 to 5.448)	0.488 (0.050 to 4.748)	0.489 (0.050 to 4.762)
**Employment status**				
Not employed		1 [Reference]	1 [Reference]	1 [Reference]
Employed		0.996 (0.331 to 2.997)	0.979 (0.323 to 2.971)	1.097 (0.362 to 3.329)
**Annual income, $**				
<20,000		1 [Reference]	1 [Reference]	1 [Reference]
20,000–39,999		0.665 (0.229 to 1.931)	0.664 (0.228 to 1.931)	0.638 (0.217 to 1.881)
≥40,000		1.135 (0.244 to 5.276)	1.182 (0.252 to 5.550)	1.234 (0.265 to 5.746)
**Marital status**				
Not married		1 [Reference]	1 [Reference]	1 [Reference]
Married		1.889 (0.774 to 4.612)	1.864 (0.761 to 4.564)	1.925 (0.781 to 4.745)
**Enabling Factors**				
**Metropolitan statistical area**				
No			1 [Reference]	1 [Reference]
Yes			1.062 (0.296 to 3.816)	1.055 (0.290 to 3.834)
**Census region**				
Northeast			1 [Reference]	1 [Reference]
Midwest			2.541 (0.466 to 13.846)	2.498 (0.454 to 13.747)
South			1.473 (0.467 to 4.652)	1.465 (0.460 to 4.669)
West			0.991 (0.307 to 3.200)	1.021 (0.312 to 3.337)
**Need Factors**				
**Perceived health status**				
Excellent/very good/good				1 [Reference]
Fair/poor				0.909 (0.342 to 2.411)
**Perceived mental health status**				
Excellent/very good/good				1 [Reference]
Fair/poor				1.004 (0.253 to 3.983)
**Help with ADL**				
No				1 [Reference]
Yes				0.226 (0.027 to 1.918)
**Help with IADL**				
No				NA
Yes				NA
**Coordination, OR (95% CI)**				
**Race/ethnicity**				
Non-Hispanic white	1 [Reference]	1 [Reference]	1 [Reference]	1 [Reference]
Non-Hispanic black	0.990 (0.752 to 1.305)	1.045 (0.786 to 1.390)	0.954 (0.711 to 1.282)	0.934 (0.695 to 1.256)
Hispanic	0.941 (0.717 to 1.235)	1.009 (0.751 to 1.355)	1.106 (0.811 to 1.509)	1.097 (0.803 to 1.499)
Non-Hispanic Asian	0.785 (0.509 to 1.209)	0.768 (0.494 to 1.194)	0.908 (0.574 to 1.437)	0.928 (0.586 to 1.469)
Non-Hispanic other	0.600 (0.331 to 1.087)	0.628 (0.345 to 1.144)	0.678 (0.369 to 1.247)	0.683 (0.370 to 1.258)
**Predisposing Factors**				
**Age, y**				
18–45		1 [Reference]	1 [Reference]	1 [Reference]
46–64		1.149 (0.832 to 1.586)	1.147 (0.829 to 1.586)	1.159 (0.837 to 1.605)
>64		1.234 (0.867 to 1.758)	1.245 (0.871 to 1.778)	1.231 (0.860 to 1.762)
**Sex**				
Male		1 [Reference]	1 [Reference]	1 [Reference]
Female		0.968 (0.771 to 1.214)	0.977 (0.778 to 1.227)	0.967 (0.770 to 1.215)
**Health insurance**				
Private		1 [Reference]	1 [Reference]	1 [Reference]
Public		0.905 (0.680 to 1.204)	0.892 (0.670 to 1.188)	0.876 (0.657 to 1.169)
No insurance		0.690 (0.463 to 1.026)	0.667^b^ (0.446 to 0.997)	0.666^b^ (0.445 to 0.996)
**Education**				
No degree		1 [Reference]	1 [Reference]	1 [Reference]
High school diploma		1.284 (0.891 to 1.851)	1.271 (0.879 to 1.837)	1.290 (0.892 to 1.868)
≥Bachelor degree		1.183 (0.716 to 1.954)	1.191 (0.717 to 1.976)	1.211 (0.729 to 2.014)
Other		1.342 (0.615 to 2.930)	1.438 (0.655 to 3.159)	1.464 (0.666 to 3.217)
**Employment status**				
Not employed		1 [Reference]	1 [Reference]	1 [Reference]
Employed		1.004 (0.745 to 1.354)	1.008 (0.748 to 1.359)	1.023 (0.756 to 1.385)
**Annual income, $**				
<20,000		1 [Reference]	1 [Reference]	1 [Reference]
20,000–39,999		0.699^b^ (0.519 to 0.942)	0.729^b^ (0.540 to 0.983)	0.735^b^ (0.544 to 0.993)
≥40,000		0.787 (0.545 to 1.137)	0.846 (0.585 to 1.225)	0.854 (0.589 to 1.239)
**Marital status**				
Not married		1 [Reference]	1 [Reference]	1 [Reference]
Married		1.205 (0.956 to 1.519)	1.216 (0.963 to 1.535)	1.202 (0.950 to 1.520)
**Enabling Factors**				
**Metropolitan statistical area**				
No			1 [Reference]	1 [Reference]
Yes			0.638^b^ (0.451 to 0.902)	0.632^b^ (0.447 to 0.894)
**Census region**				
Northeast			1 [Reference]	1 [Reference]
Midwest			0.591^a^ (0.403 to 0.866)	0.580^a^ (0.396 to 0.851)
South			0.955 (0.677 to 1.346)	0.934 (0.662 to 1.319)
West			0.579^a^ (0.405 to 0.827)	0.560^a^ (0.391 to 0.802)
**Need Factors**				
**Perceived health status**				
Excellent/very good/good				1 [Reference]
Fair/poor				1.145 (0.874 to 1.498)
**Perceived mental health status**				
Excellent/very good/good				1 [Reference]
Fair/poor				0.799 (0.565 to 1.128)
**Help with ADL**				
No				1 [Reference]
Yes				1.681 (0.907 to 3.114)
**Help with IADL**				
No				1 [Reference]
Yes				0.942 (0.587 to 1.510)

Abbreviations: ADL, activities of daily living; CI, confidence interval; IADL, instrumental activities of daily living; NA, not applicable; OR, odds ratio; USC; usual source of care.

a *P* < .01.

b *P* < .05.

c *P* < .001.

Models 2 through 4 show the results of multiple regressions or multivariate logistic regressions (Table 3). The β coefficients or ORs were adjusted for covariates potentially related to the experience of primary care by sequentially adding the clusters of covariates in the following order: predisposing, enabling, and need factors. For the first contact domain, non-Hispanic Asians still had significantly higher scores (β = 0.258, 95% CI, 0.066−0.450, *P* < 0.01) after accounting for individuals’ predisposing factors. For the first contact domain, adults aged 46 to 64 years were associated with significantly lower scores (β = −0.187; 95% CI, −0.353 to −0.022; *P* < .05), adults with an annual income of $20,000 to $39,999 were associated with significantly higher scores (β = 0.138; 95% CI, 0.007−0.270; *P* < .05), and married adults (β = 0.203; 95% CI, 0.098−0.308; *P* < .001) were associated with significantly higher scores. Similar results were also found in Models 3 and 4. Moreover, enabling factors such as MSA and census region were also significantly associated with differences in first contact scores; whereas need factors were not.

For the longitudinality domain, the significant differences that were found in Model 1 were no longer apparent in Model 2 after adjusting for predisposing factors, in Model 3 after adjusting for predisposing and enabling factors, and in the fully adjusted Model 4 after accounting for all predisposing, enabling, and need factors. Only one racial/ethnic minority group, Hispanics (OR = 0.270; 95% CI, 0.083−0.833; *P* < .05), was still significantly associated with lower odds in reporting that USC provider listened in Model 2, and no negative associations were found in Models 3 and 4. The predisposing factors of being older than 64 years and female were associated with higher odds of reporting that providers listened to them. No enabling and need factors were associated with the longitudinality indicator. 

For comprehensiveness domain, the significant association between non-Hispanic others and lower odds of going to a USC for preventive health care in Model 1 were no longer significant after controlling for the confounding factors in Models 2 through 4. For the coordination domain, no significant associations between racial/ethnic groups and lower odds of a provider asking about other treatments were found after adding the clusters of predisposing, enabling, and need factors. The predisposing factors of being uninsured and being at the $20,000 to $39,999 income level, and the enabling factors of MSA area and being in the Midwest and West census region were associated with lower odds of the provider asking about other treatments.

## Discussion

Although early differences in quality of care existed when exploring racial and ethnic disparities in primary care for patients with type 2 diabetes, these differences were no longer significant after controlling for confounding factors. Other predisposing and enabling factors, such as age, annual income, marital status, insurance coverage, MSA and census region, were associated with differences in certain primary care quality domains. This finding is consistent with findings of previous research, which indicate that socioeconomic status (SES) is a stronger determinant of diabetes status and outcomes than is race/ethnicity (2,21,22).

Beginning with *Healthy People 2000*, efforts to eliminate racial/ethnic disparities have been on the US national health policy agenda (23,24). A growing body of targeted programs, initiatives, and studies have been implemented to improve quality and address disparities in health care for racial/ethnic minorities (25). Previous evidence indicates the crucial role that primary care plays in the quality and accessibility of care for patients (26). Our research showed that when variables related to SES are controlled for, racial and ethnic disparities in access to and quality of primary care among diabetes patients were reduced and disappeared. Policy makers should continue their commitment to extend primary care to patients and focus on patients with lower SES in an effort to provide equitable services to all.

When looking at differences by race/ethnicity, patterns for non-Hispanic others often mirrored those of Hispanics and non-Hispanic blacks, with primary care quality being lower for these adults than for non-Hispanic white and Asian adults. Additional research should be conducted on the composition of the “other” racial/ethnic category to draw more precise conclusions about the primary care needs of this diverse population.

Our study has limitations. First, MEPS data on primary care are self-reported and are subject to recall bias. Second, the secondary nature of the data set precluded causal inferences. Third, the study investigated the primary care experience reported by the patients rather than their health outcomes. More studies are needed to examine the associations between the process and outcome of primary care among minority patients with diabetes. Finally, our measures of primary care attributes were operationalized from MEPS rather than being researcher-initiated measures, which limited the ability to include all the major measures of primary care, especially for measures of longitudinality and comprehensiveness (7). Other measures could be selected from MEPS to investigate the effects of the 4 domains in varying ways.

Despite these limitations, this study presents relevant findings to the field and could improve the care provided to patients with diabetes. Our findings indicate that primary care quality is equitable for diabetes patients across 4 key domains of primary care. In addition to racial/ethnic disparities in health and health care, other socioeconomic stratification factors, such as minorities disproportionately having low income and vulnerable populations having higher risk of being chronically ill and disabled, may be the cause of these disparities in population health (27). Federal efforts targeted at eliminating racial/ethnic disparities in health and health care are long-standing and persistent (28). The Patient Protection and Affordable Care Act brought provisions related broadly to health insurance coverage, which would reduce SES-related disparities in insurance coverage and access to care (28). Future efforts are needed to investigate both race-based and SES-based disparities in population health and health care. Most of the evidence suggests that equitable primary care eliminates disparities (19,29,30). Next steps and future research should be undertaken to examine the role of primary care in improvements in the management of chronic diseases by reducing both race-based and SES-based disparities.
